# Temporary organ displacement coupled with image-guided, intensity-modulated radiotherapy for paraspinal tumors

**DOI:** 10.1186/1748-717X-8-150

**Published:** 2013-06-24

**Authors:** Evangelia Katsoulakis, Stephen B Solomon, Majid Maybody, Douglas Housman, Greg Niyazov, Nadeem Riaz, Michael Lovelock, Daniel E Spratt, Joseph P Erinjeri, Raymond H Thornton, Yoshiya Yamada

**Affiliations:** 1 Departments of Radiation Oncology, Memorial Sloan-Kettering Cancer Center, 1275 York Avenue, New York, NY 10065, USA; 2 Department of Radiology, Memorial Sloan-Kettering Cancer Center, 1275 York Avenue, New York, NY 10065, USA; 3 Department of Medical Physics, Memorial Sloan-Kettering Cancer Center, 1275 York Avenue, New York, NY 10065, USA; 4 The Harold Leever Regional Cancer Center, Waterbury, CT, USA

**Keywords:** Organ displacement, Radiosurgery, Image guidance

## Abstract

**Background:**

To investigate the feasibility and dosimetric improvements of a novel technique to temporarily displace critical structures in the pelvis and abdomen from tumor during high-dose radiotherapy.

**Methods:**

Between 2010 and 2012, 11 patients received high-dose image-guided intensity-modulated radiotherapy with temporary organ displacement (TOD) at our institution. In all cases, imaging revealed tumor abutting critical structures. An all-purpose drainage catheter was introduced between the gross tumor volume (GTV) and critical organs at risk (OAR) and infused with normal saline (NS) containing 5-10% iohexol. Radiation planning was performed with the displaced OARs and positional reproducibility was confirmed with cone-beam CT (CBCT). Patients were treated within 36 hours of catheter placement. Radiation plans were re-optimized using pre-TOD OARs to the same prescription and dosimetrically compared with post-TOD plans. A two-tailed permutation test was performed on each dosimetric measure.

**Results:**

The bowel/rectum was displaced in six patients and kidney in four patients. One patient was excluded due to poor visualization of the OAR; thus 10 patients were analyzed. A mean of 229 ml (range, 80–1000) of NS 5-10% iohexol infusion resulted in OAR mean displacement of 17.5 mm (range, 7–32). The median dose prescribed was 2400 cGy in one fraction (range, 2100–3000 in 3 fractions). The mean GTV D_min_ and PTV D_min_ pre- and post-bowel TOD IG-IMRT dosimetry significantly increased from 1473 cGy to 2086 cGy (p=0.015) and 714 cGy to 1214 cGy (p=0.021), respectively. TOD increased mean PTV D95 by 27.14% of prescription (p=0.014) while the PTV D05 decreased by 9.2% (p=0.011). TOD of the bowel resulted in a 39% decrease in mean bowel D_max_ (p=0.008) confirmed by CBCT. TOD of the kidney significantly decreased mean kidney dose and D_max_ by 25% (0.022).

**Conclusions:**

TOD was well tolerated, reproducible, and facilitated dose escalation to previously radioresistant tumors abutting critical structures while minimizing dose to OARs.

## Introduction

Radiation treatment planning and delivery methods have become increasingly conformal over the past 50 years. The evolution from classical two-dimensional (2D) approaches into customized 3D techniques has been sustained by the principle that better targeting enables accurate dose delivery to tumor with concomitant dose reduction to normal tissues. Conformal radiation was further refined with the development of dose-sculpting techniques, namely IMRT. These techniques have facilitated tumor dose escalation while simultaneously decreasing toxicity, resulting in improved outcomes. Dose intensification is especially critical for the ablation of unfavorable tumor histologies such as sarcoma, renal cell carcinoma, chordoma, and non-seminomatous germ cell carcinoma [1,2]. More recently, image-guided IMRT (IG-IMRT), with 3D imaging to verify position, has allowed delivery of greater doses per fraction with marked accuracy. There are cases, however, where the tumor is immediately adjacent to a critical structure. Temporary displacement of critical organs at risk (OAR) away from the tumor during treatment allows delivery of high-dose radiation while minimizing dose to the adjacent OAR.

Multiple techniques aimed at shifting critical structures apart from the PTV have been employed in conventionally fractionated conformal radiation. Simple maneuvers such as moderate-deep inspiration breath hold during radiation have achieved substantial internal organ displacement in the treatment of left-sided breast cancer, resulting in decreased cardiac dose [3]. Slightly more invasive techniques have been examined in attempts to physically separate the rectum from the prostate in dose-escalation strategies for prostate cancer treatment. Approaches have included collagen and hyaluronic acid injections, as well as biodegradable balloon implantation between the rectum and prostate [4,5]. A prostate rectum separation of 10 mm significantly reduced mean V70 to the rectum by 83% (p<0.05), which should reduce chronic rectal toxicity [6]. Due to lengthy treatment times of conventional fractionation of up to 9 weeks, optimal spacers have yet to be routinely incorporated into clinical practice. Another technique to decrease dose to normal tissues through tighter margins via target immobilization was the endorectal balloon (ERB) which reduced maximal tumor displacement from 4 mm to ≤ 1 mm [7]. The use of ERB has been routine in clinical practice in order to immobilize target, limit intra and inter-fractional motion, and decrease normal tissue toxicity through tighter margins.

Unlike tumors in the lung or prostate, paraspinal and sacral/pelvic tumors bear a fixed topographical relationship to the spine and pelvis. Treatment can sometimes be limited by the proximity of the bowel or kidney. Various organ displacement methods have been performed to protect critical organs from thermal injury during percutaneous image-guided radiofrequency tumor ablation including gas, fluid, or balloons [8-11]. We thus focused our efforts on manipulating the motion of critical structures at risk through temporary displacement.

Radiosurgery is an ideal platform for organ displacement due to steep dose gradients and short fractionation schemes. Seemingly small shifts in critical OARs can translate into large improvements in tumor-ablative dose delivery. In this paper, we present a cohort of patients treated with tumor-ablative radiosurgery and temporary organ displacement (TOD) for radioresistant tumors that abutted critical normal tissues.

## Materials and methods

Records of 11 consecutive patients with unfavorable tumor histologies treated with TOD and IG-IMRT between 2/10 and 6/12 were retrospectively reviewed. All patients had at least one pretreatment scan [magnetic resonance imaging (MRI) or computed tomography (CT)] that revealed close proximity of tumor and normal critical structures. All patients were evaluated for TOD by a multidisciplinary team including radiation oncologists, neurosurgeons, and interventional radiologists.

### TOD technique

In all cases, intravenous procedural conscious sedation with midazolam and fentanyl is used. Under CT guidance, a 21 G needle is used to access the potential space between the OAR and the PTV. Using the Seldinger technique, the needle is exchanged for an all-purpose drain (6–10 Fr). Catheter placement and position verification are performed under image guidance by fluoroscopy and CT. Normal saline with 5-10% iohexol is infused through the catheter in a stepwise manner of 20–50 cm^3^ boluses until an adequate displacement occurs and is confirmed radiographically. Infused fluid displaces the OAR from the PTV. The infused volume is absorbed by the body over time. After recovery in the postanesthesia care unit, the patient is transported to CT simulation.

### Radiation technique

The immobilization technique at Memorial Sloan-Kettering Cancer Center has been previously described [12]. Briefly, the Memorial Sloan-Kettering Cancer Center Body Cradle immobilization device uses pressure plates applied laterally to the pelvic bones and ribs under the arms with adjustable hand grips.

An all-purpose drain (APD) is used for TOD and is placed approximately 4 hours prior to simulation. CT scan is performed prior to CT simulation and TOD positioning is re-assessed. Additional contrast material during simulation is introduced through the APD on a case-by-case basis. Typically, a 20–50 cm^3^ bolus of NS iohexol solution is incrementally introduced and satisfactory TOD is confirmed by CT. After CT simulation, the patient is discharged upon receiving catheter care education and is instructed to administer 10 cm^3^ saline flushes every 24 h. Gross tumor volume (GTV) is contoured using all available imaging information from MRI and CT. Planning target volume (PTV) consisted of clinical target volume plus a 3D margin of 3 mm. The contrast-infused TOD space enables visualization of OARs that are contoured separately.

A single fraction of 2400 cGy is prescribed for each lesion. An IG-IMRT plan is then designed to treat the PTV to the prescription dose. Our institutional dose constraints were used with maximal IG-IMRT point dose to the bowel and rectum limited to 16 Gy in one fraction. The kidney constraint was V10 Gy limited to 35% of total kidney volume in one fraction or V15 Gy limited to 35% in 3 fractions. Patients were treated within 36 h of CT simulation. Once we became comfortable with treatment delivery and consistency of TOD, we were able to estimate the degree of critical organ shifts that a TOD would permit on the planning CT simulation and subsequently confirm these shifts with actual TOD prior to treatment delivery on the same day.

### Radiation treatment

Patients were treated within 36 h of catheter placement to maintain flowing drainage and decrease the risk of catheter blockage. For treatment delivery, good catheter flow is confirmed and the patient is positioned in the cradle on the treatment couch, and cone-beam CT is obtained. The same amount of contrast material used during initial catheter placement is again introduced through the APD until satisfactory TOD is achieved. After treatment is completed, the catheter is removed by interventional radiology.

### Dosimetric comparison: radiation planning without TOD

In patients who underwent CT simulation with TOD, diagnostic CT scans prior to TOD placement were fused with CT simulation scans. OARs without displacement were contoured for all patients on the fused planning scans. A total of 10 IG-IMRT plans were generated for all patients using the modified OAR with identical prescriptions and normal tissue constraints as the TOD treatment plans. Differences in dosimetry were quantified. Dose-insufficiency measures were compared by examining GTV D_min_, PTV D_min_, and D95. Dose homogeneity was evaluated by examining D05. Dose to critical structures was examined.

To determine the statistical significance of any differences between the two groups, the outcome of the study was simulated numerically using a permutation test on each of the dosimetric measures. For each patient, the value of the measure was selected randomly for the TOD and no-TOD values observed. The process is repeated for each patient, and a sum statistic is created from the sum of the randomly selected measures. A distribution of sum statistics was generated by repeating the procedure 10^6^ times. The *p* value was calculated from the proportion of the distribution less than or equal to the sum statistic seen in the data. Because the test being used is two-tailed, this proportion was doubled to obtain the *p* values [13].

## Results

Between 2010 and 2012, 11 consecutive patients underwent IG-IMRT with TOD. The first patient was excluded from this analysis secondary to lack of contrast in injected saline during CT simulation, resulting in poor visualization of the OAR, leaving 10 patients. All patients had histologies traditionally thought to be radioresistant (Tables 1 and 2). The median age was 56 years (range, 20–80) with 3 women and 7 men. OARs included bowel/rectum (n=6) and kidney (n=4).

**Table 1:** Patient characteristics, temporary organ displacement of the bowel

**Patient**	**Primary**	**Age**	**Sex**	**Location**	**Displacement (mm)**	**Dose/fxn**
Bowel						
1	Chordoma	80	M	S3-4	10	2100 cGy/1
2	Chordoma	57	M	S3-5	31	2400 cGy/1
3	Chordoma	76	F	S3	21	2400 cGy/1
4	Sarcoma	76	F	S3	22	2400 cGy/1
5	Chordoma	48	M	S2-4	32	2400 cGy/1
6	Chordoma	54	F	S3-Coccyx	15	2400 cGy/1
					Mean 21.8	

**Table 2:** Patient characteristics, temporary organ displacement of the kidney

**Patient**	**Primary**	**Age**	**Sex**	**Location**	**Displacement (mm)**	**Dose/fxn**
1§	Renal Cell Ca	20	M	L3	7	2400cGy/1
2	NSGCT	39	M	L1	17	2850cGy/3
3*	Sarcoma	52	M	L2	7	3000cGy/3
4	NSGCT	61	M	L2-3	13	2400cGy/1
					Mean 11	

§ Patient 1 had a solitary kidney.

* Patient 3 had a horseshoe kidney.

TOD was achieved with a single APD (mean 8.5 F; range, 6–10 F) in 9 patients. One patient had an occluded catheter on treatment day, exchanged for a 12 × 40 mm balloon catheter used for displacement. The mean volume of NS solution with 5-10% iohexol used for organ displacement was 229 mL (range, 80–1000 mL). One patient had particularly difficult anatomy for which TOD catheter placement by interventional radiology was not possible. The technical success of TOD placement was thus 92% (11/12)**.** All eleven patients who had successful TOD placement went on to receive radiation treatment. The mean total procedural time for TOD placement including verification was 87.5 minutes (range, 60–150). There were no complications.

**Figure 1:**
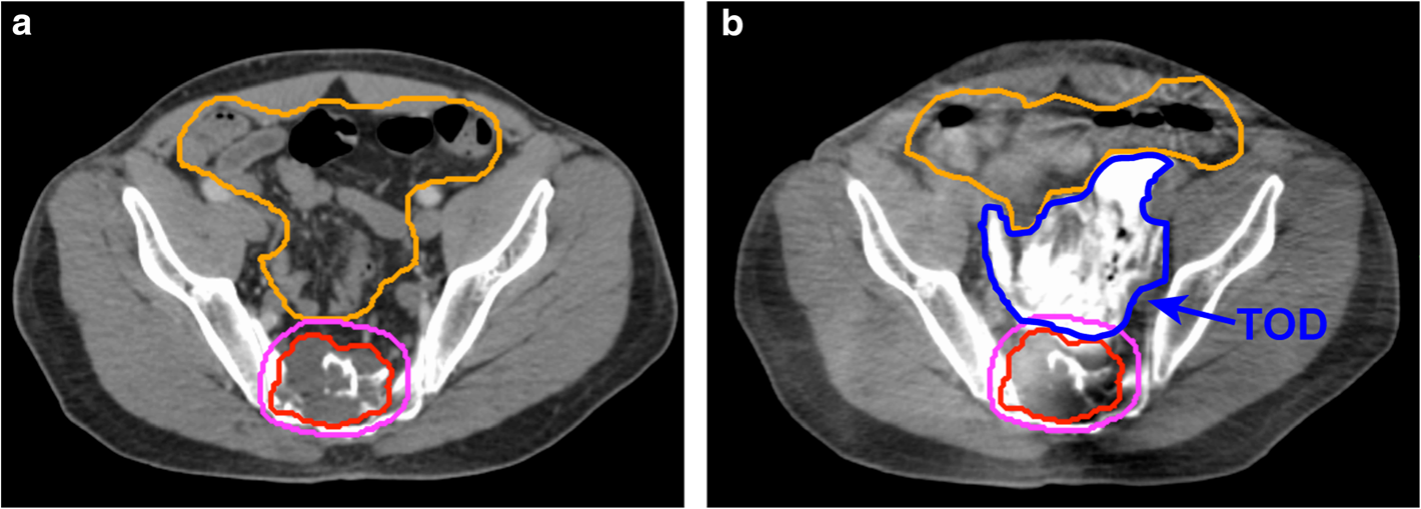
**Axial imaging of a sacral chordoma treated to 2400c Gy.** (**a**) Baseline computed tomography (CT) scan revealing pelvic sacral tumor target adjacent to bowel at S2-4. (**b**) Cone-beam CT confirming temporary organ displacement (TOD) bowel. TOD is well visualized as a hyperdense region (dark blue) between the planning target volume (pink) and bowel (orange). CBCT enables calculation of dose delivered to the critical structures.

**Figure 2:**
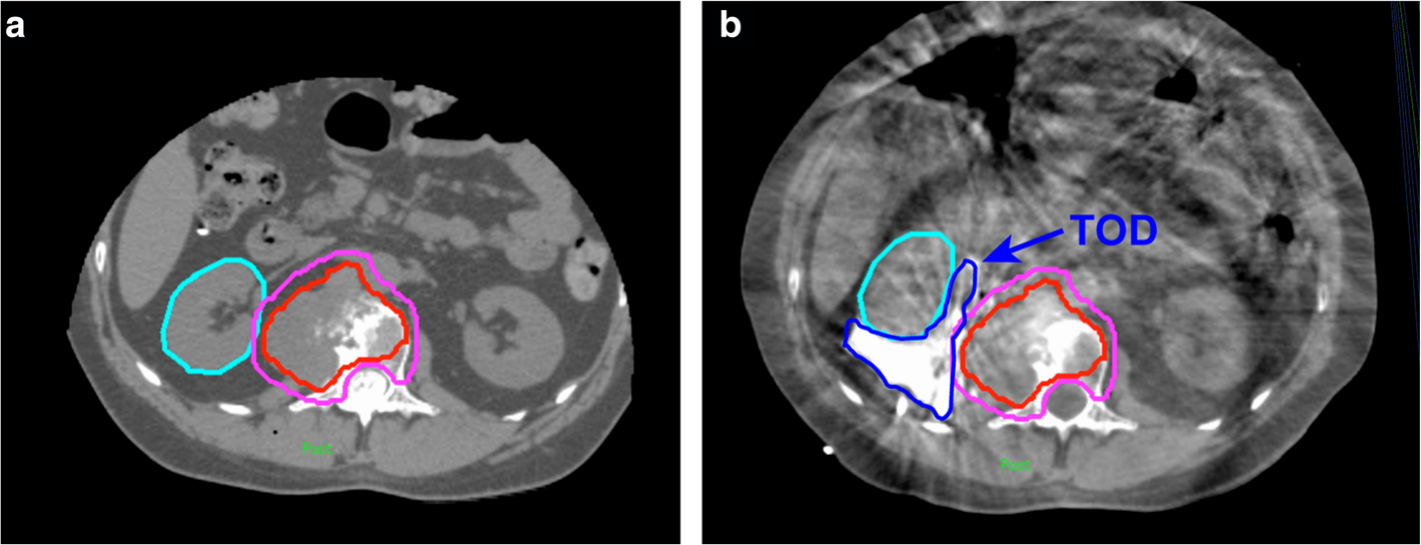
**Axial imaging of paraspinal non-seminomatous germ cell tumor treated to 2850 cGy.** (**a**) Baseline computed tomography (CT) scan revealing paraspinal target lesion adjacent to the kidney at L1. (**b**) Cone-beam CT confirming TOD kidney.

On axial CT, the TOD was well visualized as a hyperdense region between the PTV and the bowel (Figure 1b) or kidney (Figure 2b). Mean organ displacement measured at the region of the smallest separation between PTV and OAR prior to TOD was 17.5 mm. The displacement of OAR was consistently greater than or equal to the measured displacement throughout the length of the PTV.

### TOD of the bowel resulted in improved PTV coverage

**Figure 3:**
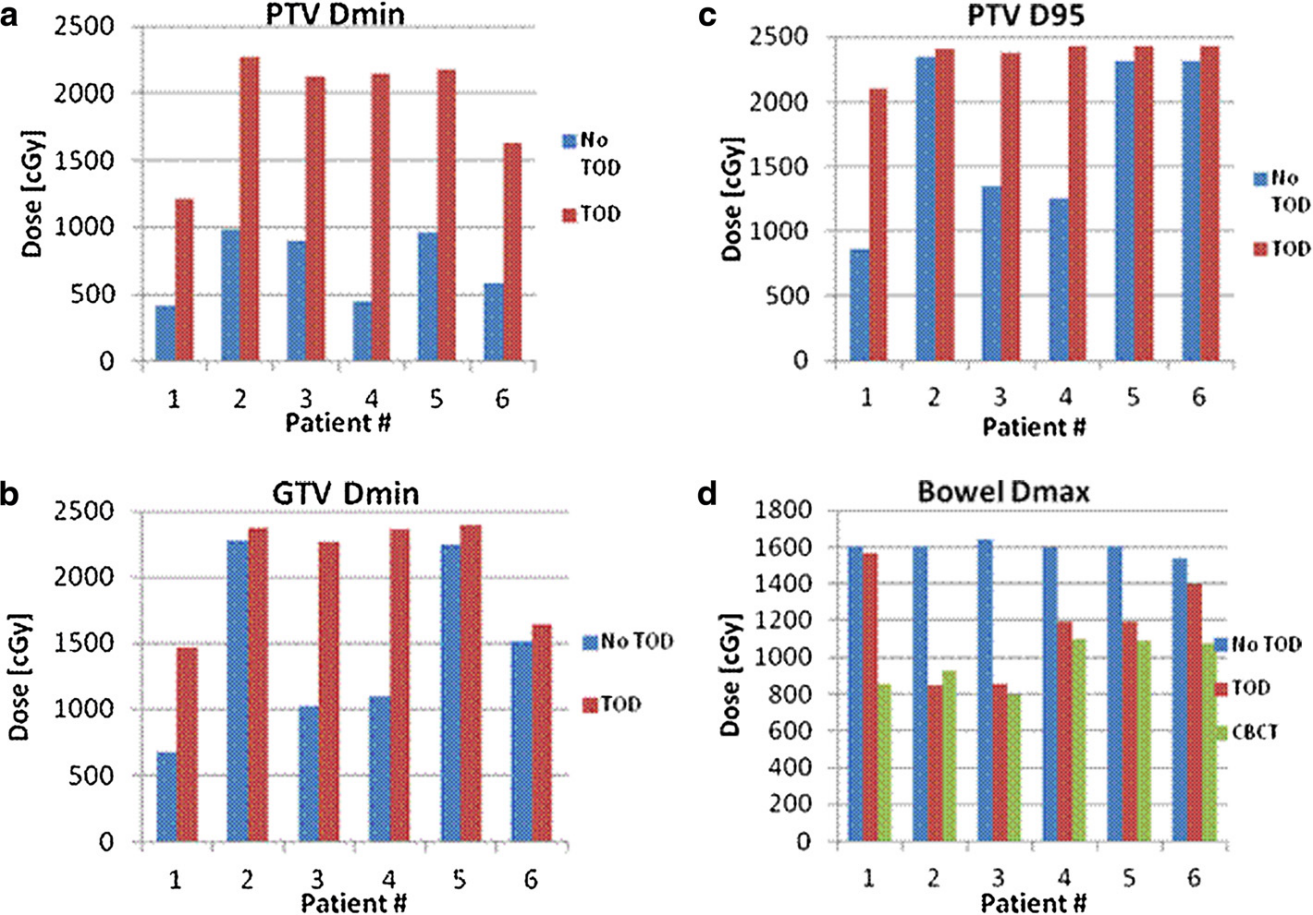
**Dosimetric outcomes with temporary organ displacement (TOD), bowel.** The bar graphs reflect pre-TOD dosimetry (blue), post-TOD dosimetry (red), and dose delivered by cone beam computed tomography (green). (**a**) Planning target volume (PTV) Dmin, (**b**) gross tumor volume (GTV) Dmin, (**c**) PTV D95, (**d**) bowel D_max_.

**Figure 4:**
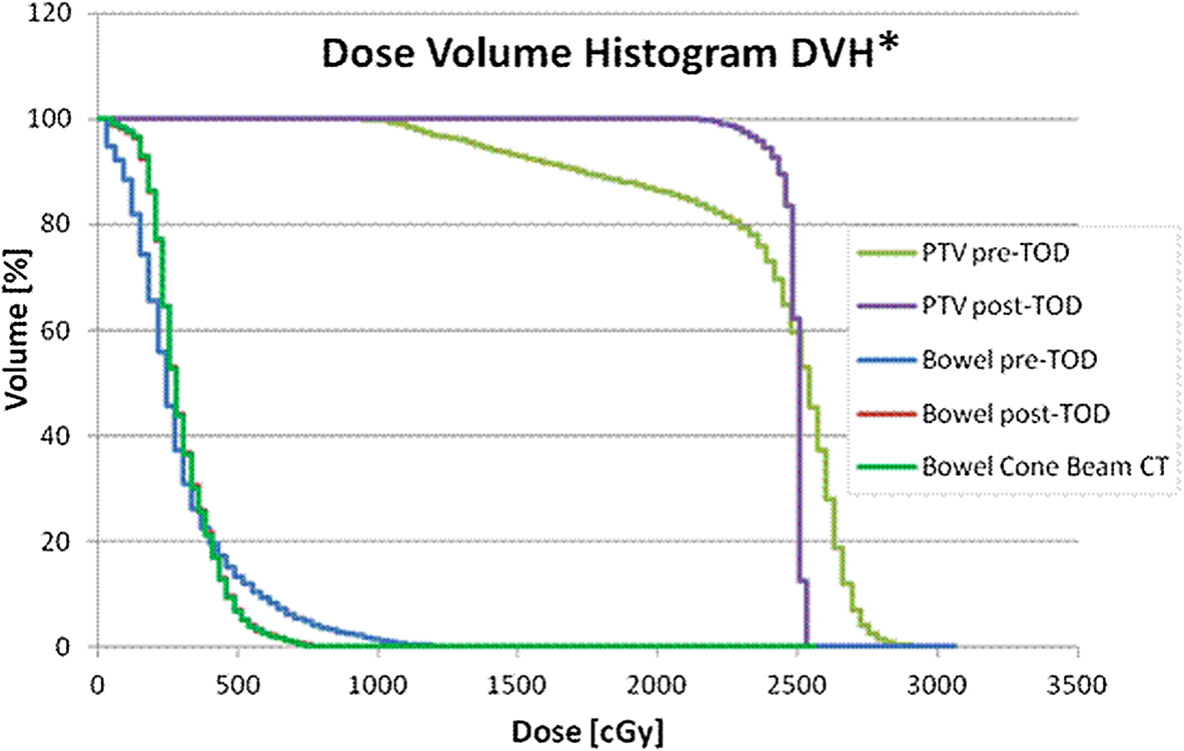
**Dose volume histogram (DVH) reflecting dosimetric advantages with temporary organ displacement (TOD), bowel.** The planning target volume (PTV) D95 increased from 1351 cGy to 2372 cGy while the D05 decreased from 2711 cGy to 2516 cGy. The GTV Dmin increased from 1025 cGy to 2262 cGy. The pre-TOD and post-TOD bowel D_max_ were 1600 cGy and 743 cGy, respectively. Dose delivered to bowel by cone-beam computed tomography was 794 cGy. *DVH corresponds to patient 3 in Table 1.

Both GTV D_min_ and PTV D_min_ were compared pre- and post- bowel TOD as shown in Figure 3a-b. The mean GTV D_min_ and PTV D_min_ pre- and post-TOD significantly increased from 1473 cGy to 2086 cGy (*p* = 0.015) and 714 cGy to 1214 cGy (*p* = 0.021), respectively. The mean PTV D95 prior to TOD also improved from 1737 cGy to 2359 cGy, or an increase of 27.14% of prescription (*p* = 0.014) (Figure 3c). A sample DVH revealing the dosimetric advantages of bowel TOD is shown in Figure 4. In addition to providing improved PTV coverage, TOD improved dose homogeneity. The mean PTV D05 significantly decreased from 2672 cGy to 2456 cGy, or 9.2% of prescription (*p* = 0.011). TOD of the kidney did not result in significant changes to PTV coverage or dose homogeneity.

### TOD resulted in reduced dose to OAR confirmed by cone-beam CT

The bowel D_max_ was compared pre-TOD with actual dose delivered by cone-beam CT as shown in Figure 3d. Prior to TOD of the bowel, the IG-IMRT plans resulted in a mean bowel D_max_ of 1596 cGy. The actual mean D_max_ calculated from cone-beam CT with TOD was 974 cGy. The 39% decrease in mean bowel D_max_ with TOD was significant (*p* = 0.008).

**Figure 5:**
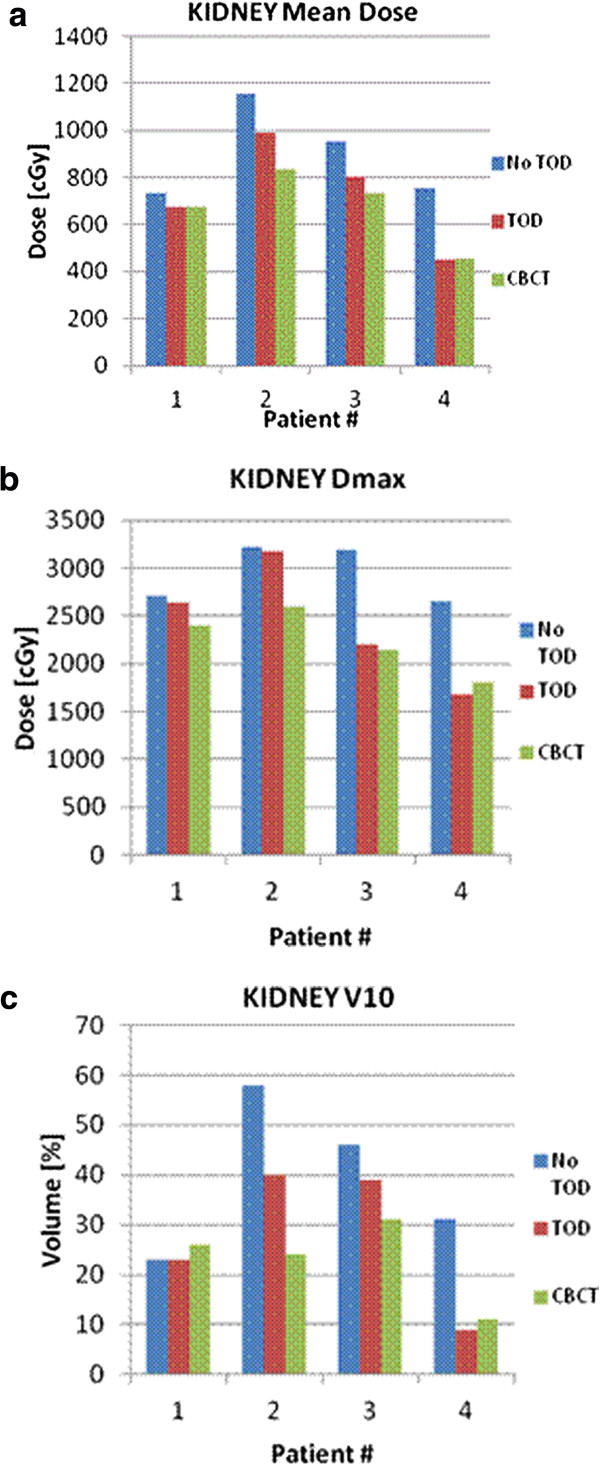
**Dosimetric outcomes with temporary organ displacement (TOD), kidney.** (**a**) Adjacent kidney mean dose. (**b**) Adjacent kidney D_max_. (**c**) Adjacent kidney V10.

The adjacent mean kidney dose, D_max_^,^ and V10 were also compared pre-TOD with cone-beam CT (Figure 5a-c). Mean kidney dose and D_max_ significantly decreased with TOD from 898 cGy to 676 cGy (*p* = 0.022) and 2942 cGy to 2235 cGy (*p* = 0.023), respectively. Mean kidney V10 decreased from 31% to 11% (*p* =0.25).

## Discussion

Unfavorable tumor histologies require tumor ablative doses to maximize local control. Image-guided techniques have enabled conformal high-dose radiation in multiple disease sites such as lung, brain, and spine. Over the past few years, we have encountered patients with unique anatomy not amenable to radiosurgery. Specifically, the critical OARs are alongside the tumor target and hinder safe delivery of radiation. To our knowledge, this is the first and only series in the radiosurgery literature that introduces a novel TOD technique into treatment planning and high-dose radiation delivery.

In this series, the mean displacement with the TOD was greater in the bowel (21.8 mm) than the kidney (11 mm). These are substantial shifts considering that the maximum dose gradient with IMRT is 10% per millimeter, thereby enabling high-dose therapy and maximizing local control. It is well established that the excellent local control rates observed for spinal metastases are dose dependent and histology independent with high-dose single fraction IGRT, reportedly >90% at 15 months. Moreover, GTV D_min_, PTV D_min_, and D95 are useful measures of dose insufficiency and ultimately local failure. In a study examining local failures after high-dose single-fraction IGRT for spinal metastases by Lovelock et al., GTV D_min_<15 Gy predicted for a 16% local failure rate compared with no failures with GTV D_min_ ≥15 Gy [14]. In this series, TOD of the bowel significantly increased both the mean GTV D_min_ and PTV D_min_ from 1473 cGy to 2086 cGy (*p* = 0.015) and 714 cGy to 1214 cGy (*p* = 0.021), respectively.

In addition to significantly improving PTV coverage, TOD of the bowel significantly reduced mean rectal and bowel D_max_ by 39% to 974 cGy (*p* = 0.008). Every organ maintains an individual dose-volume response pattern largely influenced by organ architecture. The bowel comprises a chain of functional units, with serial-like organ behavior. Late bowel injury may manifest as obstruction, bleeding, ulceration, fistula, persistent diarrhea, and perforation generally occurring weeks to months post-radiation [15]. While dose-volume parameters have been extensively reported and validated in both the bowel and rectum in the setting of conventional radiation, consensus guidelines are in development for high-dose IGRT. In the single-fraction stereotactic body radiotherapy (SBRT) study for pancreatic cancer to 25 Gy by Chang et al., <50% of the duodenum received >12.5 Gy and the 50% isodose line was not permitted to encompass the entire luminal wall. The crude rate of grade ≥3 gastrointestinal toxicity was 9% [16]. In an analysis of patients with grade 2–4 gastrointestinal toxicity following single-fraction SBRT, Murphy et al. demonstrated a dose-dependent duodenal toxicity on 16% of the treated cohort, which significantly correlated with V20 and D_max_. A D_max_ of <23 Gy versus ≥23 Gy resulted in 12% versus 49% toxicity (*p* = 0.004) [17]. The median time to toxicity was 6.3 months with a 1-year actuarial rate of grade 2–4 toxicity of 29%. Ultimately, the goals of therapy are to both maximize local control and limit toxicity. It is essential that we maintain acceptable levels of toxicity for these patients, on the order of <5%-10%. Our institutional bowel and rectal constraints are conservative, with a bowel and rectal D_max_ of 16 Gy. TOD of the bowel/rectum was effective and ensured limited dose to the bowel and rectum. None of the patients in our series developed gastrointestinal toxicity.

Not surprisingly, TOD significantly decreased dose to adjacent kidney. One patient had a single kidney while another had a horseshoe kidney. The mean kidney dose and D_max_ decreased with TOD from 898 cGy to 676 cGy (*p* = 0.022) and 2942 cGy to 2235 cGy (*p* = 0.023), respectively. The kidney has a complex organization, with cortex exhibiting parallel-organ structure while the hilum and vascular trunk maintain a serial-organ structure. Dose constraints using SBRT are lacking and not validated. In the current single-fraction spine SBRT trial (RTOG 0631), a V8.4 Gy should be <200 cm^3^ total renal cortex with grade 3 renal dysfunction as an endpoint [18]. Our institutional constraints are largely extrapolated from the liver SBRT literature with V10 Gy limited to 35% of total kidney volume in one fraction or V15 Gy limited to 35% of total kidney volume in three fractions. The subject of normal tissue constraints is compounded for patients with one kidney [19]. Any substantial decrease in dose to the kidney is meaningful.

Unlike with bowel, TOD of the kidney did not significantly improve target coverage. Possible reasons for this include less organ displacement, smaller patient numbers, and an increased amount of OARs adjacent to target. Paraspinal tumors require target dose delivery while ensuring that both the kidney and cord dose are kept below tolerance. In contrast, for tumors confined to the pelvis, bowel is often the only OAR. In the rare circumstance that a small portion of cauda is adjacent to the pelvic target, dose constraints are less conservative than cord constraints, D_max_ 18 Gy versus 14 Gy. The safe delivery of radiation is foremost in our practice and, although PTV coverage was not improved, TOD of the kidney was essential in dose reduction to critical structures. As the technique is refined, it is likely that greater displacement distance for kidney will be achieved.

The technique involves indwelling drainage catheters, similar to percutaneous endoscopic gastrostomy tubes and tenckoff catheters which are used in a various clinical applications. Indwelling catheters carry a small risk of infection with appropriate antiobiotic use, on the order of 1% to less than 3% at 2 weeks [20,21]. Moreover, infection risk is strongly correlated to the duration of catheter stay and frequent catheter hub access. The risk of infection with this technique is low since indwelling catheters were inserted for less than 36 h and infrequently accessed. In addition, NS 5-10% iohexol infusion was selected as it is generally well tolerated in the peritoneal cavity and readily absorbed. Iohexal is often used even in the presence of bowel perforation or bowel obstruction for both diagnostic and interventional radiology. Moreover, dose differences with contrast are less than 0.1% with multiple beam IMRT and clinically negligible [22].

The current study presents the feasibility and dosimetric advantages of a compelling novel technique that temporarily displaces critical structures in the pelvis and abdomen, enabling the safe delivery of tumor ablative doses.

## Conclusions

By displacing critical OAR away from the tumor, bowel and kidney doses were reduced by 25-39% and tumor dose improved by 27% with bowel TOD. TOD was well tolerated and reproducible, and facilitated dose escalation while minimizing dose to OARs. TOD is a compelling novel technique facilitating dose escalation to radio resistant tumors abutting critical structures.

## Abbreviations

2D: Two-dimensional; 3D: Three-dimensional; APD: All-purpose drain; CBCT: Cone-beam CT; CT: Computed tomography; ERB: Endorectal balloon; GTV: Gross tumor volume; IG-IMRT: Image-guided intensity-modulated radiation therapy; MRI: Magnetic resonance imaging; NS: Normal saline; OAR: Organs at risk; PTV: Planning target volume; PTV D95: ; RTOG: Radiation Therapy Oncology Group; SBRT: Stereotactic body radiotherapy; TOD: Temporary organ displacement.

## Competing interests

Yoshiya Yamada: Consultant Varian Medical Systems, Speakers Bureau Institute for Medical Education. The authors declare that they have no competing interest.

## Authors’ contributions

EK YY SS MM DH NR ML made substantial contributions to concept and design of the data. SS MM JE RT performed TOD techniques as described. GN ML made substantial contributions to dosimetric analysis. EK DH DS MM performed acquisition of the data. All authors read and approved the final manuscript.
